# Presence of anti-*Leishmania infantum* antibodies in sheep (*Ovis aries*) in Spain

**DOI:** 10.1007/s11259-023-10221-y

**Published:** 2023-10-11

**Authors:** Sergio Villanueva-Saz, María Eugenia Lebrero, Alba Solsona, Juan José Ramos, Marta Ruíz de Arcaute, Héctor Ruíz, María D. Pérez, José María Bello, Maite Verde, Aurora Ortín, Diana Marteles, Antonio Fernández, Alex Gómez, Michele Trotta, Delia Lacasta

**Affiliations:** 1 Clinical Immunology Laboratory, Veterinary Faculty, University of Zaragoza, Zaragoza, 50013 Spain; 2 Department of Animal Pathology, Veterinary Faculty, University of Zaragoza, Zaragoza, Spain; 3 Instituto Agroalimentario de Aragón-IA2 (Universidad de Zaragoza-CITA), Zaragoza, Spain; 4 Department of Animal Production and Sciences of the Food, Veterinary Faculty, University of Zaragoza, Zaragoza, Spain; 5 NANTA SAU, Ronda de Poniente 9, Tres Cantos, Spain

**Keywords:** Antibodies, ELISA, *Leishmania*, Serology, Sheep, Spain

## Abstract

Sandflies are the primary transmission vector for *Leishmania* spp parasite in endemic regions. The role of other animals, different from the dog, is under discussion in the leishmaniosis endemic countries. A limited number of reports have been published on the possible role of livestock in European countries for *Leishmania* maintenance and diffusion. The aim of the present study was to perform a serosurvey on sheep in areas of Spain that are endemic for zoonotic leishmaniosis and establish the possible role of sheep regarding *Leishmania infantum* infection in endemic areas. Three hundred and two serum samples were obtained from sheep and were evaluated for serological survey to detect *L. infantum* by using the in-house ELISA technique. Twenty-eight out of the 302 samples included in this study, were positive for *L. infantum* antibodies (9.27%). In the present study, a significant association was found between adult age and seropositivity (p = 0.006) and female gender and seropositivity (p = 0.02). This association has not been previously described in other European studies related to *L. infantum* infection in sheep. Our study reveals that domestic sheep in a European Mediterranean country are exposed to *L. infantum*. To our knowledge, this study demonstrates the presence of seropositive sheep in different regions of Spain for the first time. Further epidemiological studies focus on evaluating the rural cycle of this parasite to know if livestock could act as a potential reservoir are needed.

## Introduction

*Leishmania* parasites are transmitted through the bites of infected female phlebotomine sandflies in endemic regions. Some 70 animal species, including humans, can be the source of *Leishmania* parasites (WHO 2023). According to WHO, the prevention and control of the spreading of leishmaniasis are complex and require many tools: early diagnosis, vector control, effective disease surveillance, control of animal reservoir hosts, social mobilisation and strengthening partnerships (WHO 2023). A combination strategy is crucial for the effective reduction of the rate of infection in the interested countries. The control of animal reservoir hosts is complex and should be tailored to the local situation. *Leishmania donovani* complex spp. infect humans as well as different mammalian species (Kushwaha et al. 2022).

The role of other animals, different from the dog, is under discussion in the leishmaniosis endemic countries. An essential role in maintaining infection was represented by hares in Spain (García et al. 2014). Under discussion is the role of cats in these years in European countries of the Mediterranean basin (Alcover et al. 2021). A limited number of reports have been published on the possible role of livestock (cattle, sheep, goat, donkey, and horse) in European countries for *Leishmania* maintenance and diffusion (Cardoso et al. 2021). Up to now, information on the role of sheep and leishmaniosis is limited globally; nevertheless, available data suggests that sheep may have a role in the epidemiology of *Leishmania* spp. due to the asymptomatic nature of infection in this species (Mukhtar et al. 2000; Portús et al. 2002; Rohousova et al. 2015; Han et al. 2018).

The aim of the present study was to perform a serosurvey on sheep in areas of Spain that are endemic for zoonotic leishmaniosis and establish the possible role of sheep regarding *Leishmania infantum* infection in endemic areas.

## Materials and methods

### Animals and sample collection

Three hundred and two residual serum samples were obtained from different sheep for diagnostic purposes at the Veterinary Faculty of Zaragoza (41°39′24.6276″ N, 0°52′45.912″ W). Based on an expected seroprevalence of 10% (the canine seroprevalence in Spain) (Baxarias et al. 2023), an accepted 5% deviation from the true prevalence and a confidence level of 95%, the sample size necessary to estimate the seroprevalence was calculated to be 139 animals (De Blas 2006). It is essential to highlight that these animals belong to the university farm. In the Ruminant Clinical Service (SCRUM), a small healthy, productive flock is maintained, and many referred cases are received from different farms in the northwestern area of Spain. The sera come from animals that arrive at the SCRUM as clinical cases with different diseases. They all come from sheep farms in the area of influence of the Faculty of Veterinary Medicine in Zaragoza: Aragon, the Valencian Community, the Basque Country and Navarra. All the animals come from commercial farms with a semi-intensive production system, in which they for graze an essential part of the year.

The samples were collected in different periods from autumn 2020 to winter 2022: Spring (n = 3), Summer (n = 119), Autumn (n = 69), and Winter (n = 111). During 2020, 171 samplings were performed: 3 in spring, 119 in summer, 48 during autumn, and 1 in winter. During 2021, 111 samplings were performed: 1 in autumn and 110 in winter. In 2022, 20 samplings were performed during autumn. Related to the breed, most of the sheep were purebred sheep represented by Rasa Aragonesa (n = 120), Latxa (n = 9), Manchega (n = 4), Roya Bilbilitana (n = 4), Aranesa (n = 3), Ripollesa (n = 3) Merina (n = 2), Assaf (n = 1), Lacaune (n = 1), and Maellana (n = 1). The remaining sheep were classified as mixed-breed sheep (n = 154).

Serum samples were obtained of each sheep once the referred case was received in the SCRUM facilities. Serum samples were collected associated with routine healthcare check-ups from autumn 2020 to winter 2022. The separated residual sera were stored at − 35 °C until they were processed.

Before sampling, information was obtained about each animal regarding age (lambs (< 12 months), adults (from ≥ 1 year to ≤ 6 years), or seniors (> 6 years), gender, elevation of farm location (mountains with an altitude between 792 and 1171 m, semi-mountains with an altitude between 208 and 685 m, and flat areas, with an altitude between 80 and 181 m) and the season of the year in which the blood was taken. In addition, a complete physical examination was carried out to detect the presence of potential skin lesions compatible with clinical leishmaniosis because dermatological lesions are the most common clinical signs detected in animals due to *L. infantum* (Cardoso et al. 2021).

Finally, infected sheep by some other pathogens (n = 25) were included to evaluate cross-reactivity and diagnostic specificity of the antibody test in this study. For this purpose, the pathogens included were the most prevalent in Spain, such as *Anaplasma ovis* (n = 5), *Coxiella burnetii* (n = 5), *Babesia ovis* (n = 5), *Babesia motasi* (n = 5) and *Theileria ovis* (n = 5). These serum samples were from the serum bank of the Clinical Immunology Laboratory, Veterinary Faculty, University of Zaragoza and a private laboratory.

### Detection of *L. infantum* antibodies by a quantitative ELISA

The ELISA was performed on all sera as described previously (Alcover et al. 2021), with some modifications. For the in-house ELISA (sensitivity of 99.37% and specificity of 97.50%), the crude antigen (strain MHOM/FR/78/LEM75 belonging to *L. infantum* zimodeme MON-1) was adjusted to a concentration of 20 µg/ml with phosphate-buffered saline (PBS). Briefly, each plate was coated with 100 µl/well of the 20 µg/ml antigen solution in 0.1 M carbonate/bicarbonate buffer and incubated overnight at 4 ºC. A 100-µl aliquot of sheep sera, diluted 1:100 in PBS containing 0.05% Tween 20 (PBST) and 1% dry skimmed milk (PBST-M) as a blocking agent, was added to each well. The plates were incubated for 1 h at 37 °C in a moist chamber. After washing the plates for 3 min 3 times with PBST followed by 1 wash with PBS for 1 min, 100 µl of Protein A/G conjugated to horseradish peroxidase (Thermo Fisher Scientific, Waltham, Massachusetts, USA) diluted 1:10000 in PBST-M was added per well. The standardisation of the ideal concentration of serum dilution and conjugated dilution was based on a previous case report of leishmaniosis in a small ruminant (Ruiz et al. 2023). The plates were incubated for 1 h at 37 °C in the moist chamber and were washed again with PBST and PBS as described above. The substrate solution (ortho-phenylene-diamine) and stable substrate buffer (Thermo Fisher Scientific, Waltham, Massachusetts, USA) was added at 100 µl per well and developed for 20 ± 5 min at room temperature in the dark. The reaction was stopped by adding 100 µl of 2.5 M H2SO4 to each well. Absorbance values were read at 492 nm in an automatic microELISA reader (Microplate Photometer Biosan Hipo MPP-96, Riga, Latvia). As a positive control, each plate included three serum samples, including a sick seropositive dog, a sick seropositive cat and a sick seropositive goat diagnosed with clinical leishmaniosis (Ruiz et al. 2023). Optical density (OD) units of the positive controls were > 1.1 OD units. By contrast, serum from healthy, non-infected sheep was used as a negative control with a value < 0.10 OD units. The same positive and negative sera were used for all assays, and the plates with an inter-assay variation greater than 10% were discarded. All samples were run in duplicate. The cut-off was set to 0.38 OD units (mean + 3 standard deviations (SD) of values from 90 sheep from non-endemic areas such as northern Spain), and results above this value were considered positive. These 90 sheep were classified as healthy according to Leishvet guidelines (Solano-Gallego et al. 2011; Pennisi et al. 2015). This classification was based on a complete physical examination and the absence of clinicopathological abnormalities detected by routine red blood cell count (Idexx Procyte Dx, Westbrook, ME, USA), clinical chemistry (AmiShield, Protect Life International Biomedical Inc. Taiwan), urinalysis and serum protein electrophoresis by agarose gel electrophoresis system (Hydragel Kit 1–2, Sebia, Evry, France). Laboratory parameters of these animals were not considered altered because they were within the reference intervals.

### Statistical analysis

Data collected for the entire population were analysed using descriptive statistics. Univariate analysis of categorical data was performed to determine possible associations between *L. infantum* seropositivity and the following variables: age, gender, the elevation of farm location, season, and collection year. The significance of this difference was assessed using the Fisher’s exact test or Chi-square. A *p ≤* 0.05 was considered significant. The SPSS v.22 software (SPSS Inc., Chicago, USA) was used.

## Results

### Animal studied

All the tested sheep (n = 302; 205 females, 87 males and 10 non-determined) were from different Spanish regions including Guadalajara (n = 4), Guipuzcoa (n = 8), Huesca (n = 13), Lérida (n = 1), Teruel (n = 7) and Zaragoza (n = 269). No dermatological lesions compatible with leishmaniosis were observed in the sheep included in this study. Based on their age, the animals were classified as lambs (n = 159), adults (n = 102), or seniors (n = 41). The sheep were reared in different geographical areas, including mountains (n = 21), semi-mountains (n = 174), and flat areas (n = 107).

### Serology for *L. infantum*

**Fig. 1:**
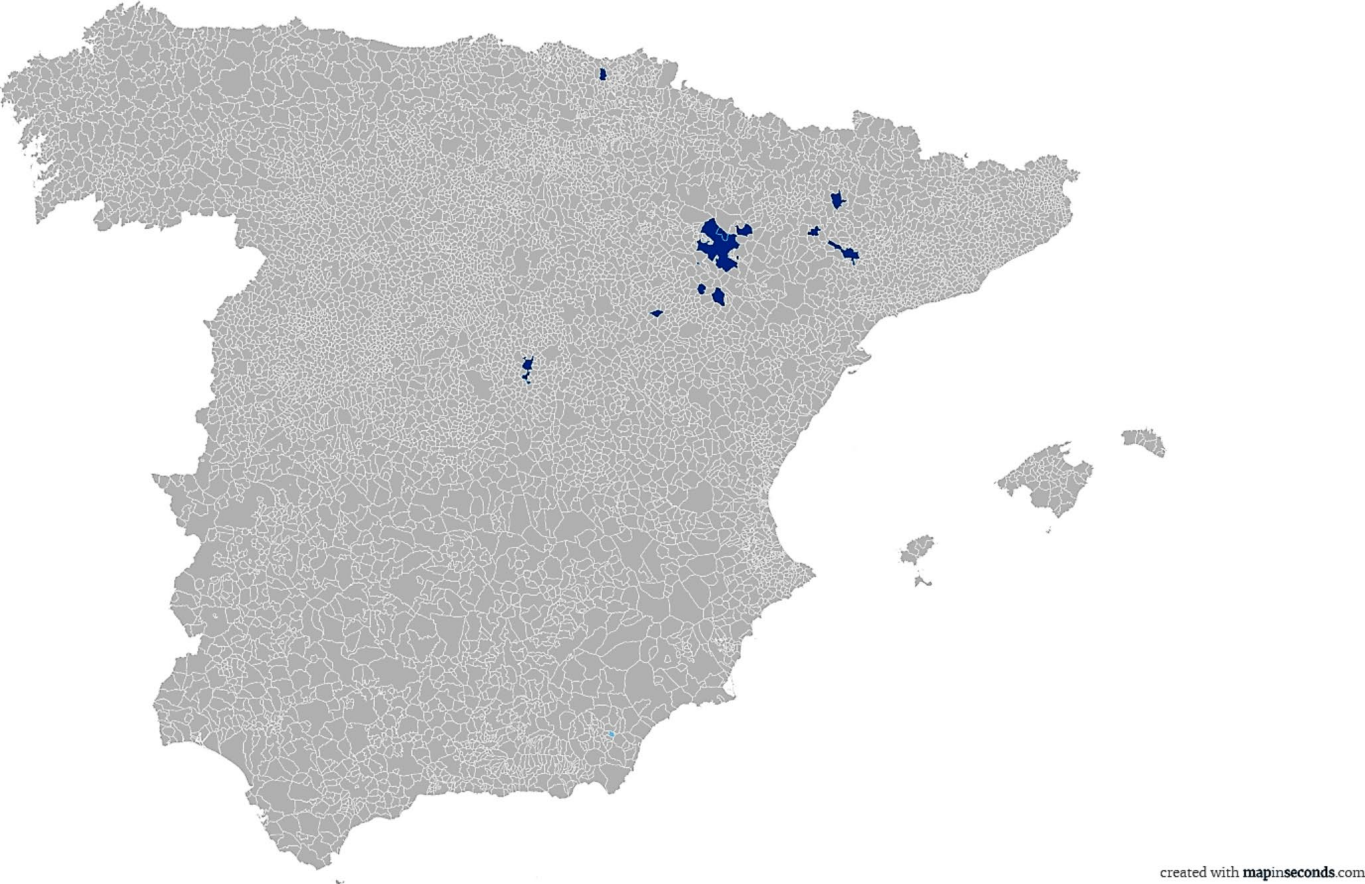
Distribution of the location of the seropositive sheep per municipalities in Spain from autumn 2020 to winter 2022. The coloured areas indicate the location of the animals

Seroprevalence of *L. infantum* infection was 9.27 (95% confidence interval [CI] 6.49–13.07%) including seropositive animals from different regions such as Guadalajara (n = 1), Guipuzcoa (n = 4), Huesca (n = 2), Lleida (n = 1), and Zaragoza (n = 20). Twenty-eight (25 females (8.28%) and 3 meales (0.99%)) out of the 302 studied samples were positive for *L. infantum* (mean ± SD: 0.464 ± 0.078) antibodies by using an in-house ELISA. The ELISA classified the remaining samples (n = 274) as negative (mean ± SD: 0.200 ± 0.073). The seroprevalence rates for each category were 4.40% for lambs (7/159), 15.84% for adults (16/101) and 12.20% for seniors (5/41). The geographical distribution of the seropositive sheep is included in Fig. 1. All the information about the seropositive samples is detailed in Table 1. Finally, none of the seropositive samples to other pathogens to evaluate cross-reaction phenomenon was positive to *L. infantum.*

**Table 1:** The twenty-eight seropositive samples from sheep evaluated in detail: by province of origin, altitude of farm location, breed, season of sampling collection, year of sampling collection, age group, age, sex, and optical density units detected by ELISA technique

PROVINCE	FARM ALTITUDE	BREED	SEASON	YEAR	AGE GROUP	AGE	SEX	Optical density units
Zaragoza	Semi-Mountains	Rasa Aragonesa	Summer	2020	Adult	5 years	Female	0.48
Zaragoza	Semi-Mountains	Rasa Aragonesa	Summer	2020	Senior	7 years	Female	0.41
Zaragoza	Mountains	Rasa Aragonesa	Summer	2020	Adult	5 years	Female	0.40
Zaragoza	Semi-Mountains	Rasa Aragonesa	Autumn	2020	Adult	6 years	Female	0.48
Zaragoza	Semi-Mountains	Rasa Aragonesa	Winter	2021	Adult	5 years	Female	0.39
Guipuzcoa	Plains	Latxa	Winter	2021	Adult	5 years	Female	0.49
Guipuzcoa	Plains	Latxa	Winter	2021	Adult	5 years	Female	0.48
Zaragoza	Semi-Mountains	Rasa Aragonesa	Winter	2021	Senior	7 years	Female	0.43
Zaragoza	Semi-Mountains	Rasa Aragonesa	Winter	2021	Adult	5 years	Female	0.52
Zaragoza	Semi-Mountains	Rasa Aragonesa	Winter	2021	Adult	5 years	Female	0.64
Huesca	Mountains	Ripollesa	Winter	2021	Adult	3 years	Female	0.73
Guipuzcoa	Plains	Latxa	Autumn	2020	Adult	4 years	Male	0.52
Zaragoza	Plains	Mixed-Breed	Summer	2020	Lamb	3 months	Female	0.50
Zaragoza	Plains	Mixed-Breed	Summer	2020	Lamb	3 months	Female	0.51
Zaragoza	Plains	Mixed-Breed	Summer	2020	Lamb	3 months	Female	0.39
Zaragoza	Plains	Mixed-Breed	Summer	2020	Lamb	3 months	Female	0.47
Zaragoza	Plains	Mixed-Breed	Summer	2020	Lamb	3 months	Female	0.39
Zaragoza	Plains	Mixed-Breed	Summer	2020	Lamb	3 months	Female	0.49
Zaragoza	Plains	Mixed-Breed	Summer	2020	Lamb	3 months	Male	0.41
Zaragoza	Semi-Mountains	Lacaune	Winter	2021	Adult	1 year	Female	0.39
Zaragoza	Semi-Mountains	Rasa Aragonesa	Winter	2021	Senior	7 years	Male	0.40
Zaragoza	Semi-Mountains	Rasa Aragonesa	Winter	2021	Senior	7 years	Female	0.39
Guipuzcoa	Plains	Latxa	Winter	2021	Adult	5 years	Female	0.49
Lleida	Plains	Ripollesa	Winter	2021	Adult	3 years	Female	0.73
Zaragoza	Semi-Mountains	Rasa Aragonesa	Winter	2021	Adult	5 years	Female	0.52
Zaragoza	Semi-Mountains	Roya Bilbilitana	Autumn	2022	Adult	3 years	Female	0.45
Huesca	Semi-Mountains	Mixed-Breed	Autumn	2022	Senior	7 years	Female	0.54
Guadalajara	Semi-Mountains	Manchega	Autumn	2020	Adult	2 years	Female	0.46

No significant association (p > 0.05) was detected between *Leishmania* seropositivity and the year of collection, season, and elevation of farm location. However, a significant association was found between age and seropositivity (p = 0.006) and gender and seropositivity (p = 0.02).

## Discussion

The dog has been implicated as the domestic reservoir of *L. infantum* infection in European Mediterranean countries including Spain as an endemic vector-borne disease. Therefore, most of the epidemiological information is focused on dogs and cats. However, new evidence supports the potential importance of other animal species, such as ferrets and goats, where the first description of clinical cases of leishmaniosis has been published (Giner et al. 2020; Ruiz et al. 2023). The seroprevalence of *L. infantum* infection in dogs in Spain was around 10% between 2011 and 2020 (Baxarias et al. 2023). Little information is available concerning *L. infantum* and livestock in Europe. In the case of sheep, different pieces of evidence suggest that the proliferation of promastigotes of different *Leishmania* species such as *L. infantum, L. donovani, Leishmania tropica and Leishmania major* in the biphasic Novy-MacNeal-Nicolle (NNN) medium prepared using sheep blood is possible. The sheep blood is able to produce cultures in lower numbers requiring longer than 7 days to appear (Ladopoulus et al. 2015).

The epidemiological role of sheep in *L. infantum* infection has been investigated in an endemic area as it is Greece, with the absence of *L. infantum* antibodies detected by ELISA in the natural environment in 361 sheep from 34 different farms from Thessaly (Kantzoura et al. 2013). By contrast, low antibody titres were detected by Dot-ELISA in sheep in Tarragona with a seroprevalence of 11.90% (Portus et al. 2002).

In the present study, the age and gender of the sheep tested showed significant associations with *Leishmania* seropositivity. This association has not been previously described in other European studies related to *L. infantum* infection in sheep. Nevertheless, age association has been described in canine leishmaniosis, with the presence of a bimodal distribution with a peak of sick dogs with less than three years and a second peak between eight and ten years (Alvar et al. 2004). In our study, the highest number of seropositive was detected in the adult group (16/101), followed by lambs (7/159), and the senior group (5/41). In general, adult and senior sheep raised in an extensive system in areas with low agriculture productivity, and these sheep are more exposed to sandflies bites. However, the lambs are kept indoor, in livestock facilities, with less exposure to the vector. In this sense, the association of canine leishmaniosis with age has been explained by a longer exposure time of the animals with sandflies vectors (Martín-Sánchez et al. 2009). Finally, a possible explanation of the association of seropositivity with the females can be related to the high number of ewes in the study, especially in the Mediterranean breed (Ortiz 1983).

Differences to the study performed in Greece (Kantzoura et al. 2013) and the studies performed in Spain include geographic location, age of animals, type of serological technique, serum dilution and type of antigen. In the case of Kantzoura et al. 2013, samples were in a double dilution (1:200) to that of the present work (1:100) in the ELISA. No information related to the antigen is available in this serological study, however in the Spanish studies, the antigen was prepared from promastigotes of *L. infantum* (strain MHOM/FR/78/LEM 75 belonging to zymodeme MON-1). Different antigenic sources are employed in serological techniques, including soluble *leishmania* antigen and different parasite antigenic fractions such as individual recombinant proteins or small peptides containing defined antigenic determinants (Ramírez et al. 2019). The use of soluble leishmanial total extract preparation as antigen appears to be more sensitive than recombinant protein antigen for the detection of anti-*Leishmania* antibodies in dogs (Miró et al. 2008) and humans (Kühne et al. 2019).

The presence of seropositive sheep to *Leishmania* infection has been detected in other countries outside of Europe. An investigation of the zoonotic leishmaniasis outbreak in China detected the presence of *Leishmania* DNA in different livestock species, including sheep, goats, cattle, and donkeys (Gao et al. 2015). In Africa, the presence of anti-*Leishmania* antibodies against *L. donovani* was detected in donkeys, cows, and goats in Sudan. However, specific antibodies against *L. donovani* were not detected in the sheep from eastern Sudan (Mukhtar et al. 2000). The presence of antibodies against *L. donovani* by modified agglutination test (DAT) has been detected in different domestic animals in Ethiopia, such as cows, dog, donkeys, goats and finally in sheep with the lowest seropositivity rate in comparison to the remaining species included in the study (Kenubih et al. 2015). The presence of anti-*Leishmania* spp. antibodies was detected by DAT in 40.1% of sheep in Iran. In this sense, due to the coexistence of different *Leishmania* species in this country, it was necessary to perform a specific quantitative molecular technique and sequencing for the identification and detection *L. infantum* and *L. major* species (Rezaei et al. 2022).

One advisable situation in the validation of serological techniques would be the inclusion of seropositive samples for other pathogens to evaluate cross-reaction phenomenon with importance in the specificity value of the analysed technique. Little is known about which sheep pathogens could be potentially cross-reacted with anti-*Leishmania* antibodies detected by ELISA. In this sense, we have included a panel of seropositive samples of the most common vector borne disease and bacterial disease with importance in Spain. The inclusion of positive serum samples to another type of *Leishmania* species phylogenetically similar would be desirable in areas where different *Leishmania* species are present and we cannot rule out the potential of cross-reactivity with other endemic species circulating in regions of the European Mediterranean basin.

The present study reveals that domestic sheep in a European Mediterranean country are exposed to the *L. infantum* infection. This study extends to other provinces of Spain the data previously obtained on the presence of *Leishmania* seropositive sheep. Further epidemiological studies focus on evaluating the rural cycle of this parasite to know if livestock could act as a potential reservoir are needed.

## Data Availability

The data that support the findings of this study are available from the corresponding author upon reasonable request.
